# A Data Taxonomy for Adaptive Multifactor Authentication in the Internet of Health Care Things

**DOI:** 10.2196/44114

**Published:** 2023-08-29

**Authors:** Tance Suleski, Mohiuddin Ahmed

**Affiliations:** 1 School of Science Edith Cowan University Perth Australia

**Keywords:** health care, authentication, contextual data model, Internet of Health Care Things, multifactor, mobile phone

## Abstract

The health care industry has faced various challenges over the past decade as we move toward a digital future where services and data are available on demand. The systems of interconnected devices, users, data, and working environments are referred to as the Internet of Health Care Things (IoHT). IoHT devices have emerged in the past decade as cost-effective solutions with large scalability capabilities to address the constraints on limited resources. These devices cater to the need for remote health care services outside of physical interactions. However, IoHT security is often overlooked because the devices are quickly deployed and configured as solutions to meet the demands of a heavily saturated industry. During the COVID-19 pandemic, studies have shown that cybercriminals are exploiting the health care industry, and data breaches are targeting user credentials through authentication vulnerabilities. Poor password use and management and the lack of multifactor authentication security posture within IoHT cause a loss of millions according to the IBM reports. Therefore, it is important that health care authentication security moves toward adaptive multifactor authentication (AMFA) to replace the traditional approaches to authentication. We identified a lack of taxonomy for data models that particularly focus on IoHT data architecture to improve the feasibility of AMFA. This viewpoint focuses on identifying key cybersecurity challenges in a theoretical framework for a data model that summarizes the main components of IoHT data. The data are to be used in modalities that are suited for health care users in modern IoHT environments and in response to the COVID-19 pandemic. To establish the data taxonomy, a review of recent IoHT papers was conducted to discuss the related work in IoHT data management and use in next-generation authentication systems. Reports, journal articles, conferences, and white papers were reviewed for IoHT authentication data technologies in relation to the problem statement of remote authentication and user management systems. Only publications written in English from the last decade were included (2012-2022) to identify key issues within the current health care practices and their management of IoHT devices. We discuss the components of the IoHT architecture from the perspective of data management and sensitivity to ensure privacy for all users. The data model addresses the security requirements of IoHT users, environments, and devices toward the automation of AMFA in health care. We found that in health care authentication, the significant threats occurring were related to data breaches owing to weak security options and poor user configuration of IoHT devices. The security requirements of IoHT data architecture and identified impactful methods of cybersecurity for health care devices, data, and their respective attacks are discussed. Data taxonomy provides better understanding, solutions, and improvements of user authentication in remote working environments for security features.

## Introduction

### Overview

There is an emergence of research on the Internet of Health Care Things (IoHT) in the past decade as the introduction of Internet of Things (IoT) devices overseeing sensitive health care data has dynamically shaped the environment of authentication. This viewpoint explores the current challenges to the IoHT and the technologies that are being used with health care data. The data are assessed based on communication as a service between providers and their patients through various environments. Related work is reviewed with the addition of COVID-19–related environments to support an improved mapping of current IoHT architectures. These data will benefit future research and development of authentication approaches to IoHT data modeling. The purpose of the data classification model is to contextualize the attributes that make up an IoHT device’s data structure. To achieve this, we discuss these attributes as entities within the data model and how they are related to authentication security, that is, mapping the data to the distinct categories of devices and the threats that are present or emerging against those categories of IoHT devices. From the perspective of a viewpoint, we discuss adaptive multifactor authentication (AMFA) in the context of health care. The results are an evaluation of the feasibility for improved AMFA model to address security concerns with regard to IoHT methodologies.

The main contributions of the structured model of IoHT data with regard to AMFA are as follows:

1. Establishing a viewpoint of security requirements of authentication systems from a cybersecurity perspective and discussing the challenges on the domain of IoHT data
2. Establishing the architecture of IoHT data requirements, as attributes, and forming a categorization of the 4 domains within our scope: user information, working environments, device information, and use-case settings
3. Discussing and summarizing the data taxonomy of AMFA-IoHT data in relation to the current challenges and future directions

### Background

The COVID-19 pandemic has created a lot of interest in the research community to address concerns against security issues regarding the constraints and limitations of IoT devices used in health care and seeking to improve the overall organization and security posture of authentication identity management systems [1]. The technology present in current health care industries facilitates the advancement of authentication practices with a better understanding of how AMFA can improve the IoHT and the ubiquity of these existing technologies such as in Bluetooth and Wi-Fi communications [2]. Many health care services have become remotely accessible from home and on the go as organizations remediate against the high demand for their resources and the physical capabilities of their workers and assets. The IoHT has been a crucial part of increasing the scalability of these services [3]. The IoHT has helped to reduce the cost of services for both the organizations and the patients through telecommunication advances, remote treatment and monitoring of patients for health care workers, and reducing the amount of physical interaction needed between people as social distancing was enforced across many countries to combat the threat of COVID-19 [4]. The IoHT not only improves these services but also increases the capabilities of innovating new ways by which services can be delivered. Technologies can be applied to medical practices to reduce the social impact that COVID-19 has caused the world, and experimental studies show higher accuracy and confidence toward the real-world markets of these applications [5]. In authentication security, a data breach to an organization can expose both its customers such as patients in health care and organization workers such as physicians, doctors, or nurses. The cost of a data breach can be scaled depending on the size of the organization and the type of data that are affected, and the health care industry is targeted because of the large volume of sensitive information becoming digitalized. According to the 2021 IBM report on the Cost of a Data Breach, a study showed that remotely working away from the office during the pandemic led to expensive data breaches reaching an average of US $4.96 million per breach when remote working occurred, and stolen user information was the lead cause in a data breach [6]. According to the 2022 IBM report on the Cost of a Data Breach, this was the 12th year in a row where the average cost of a data breach had increased from the previous year, showing an average cost of US $4.35 million up from US $4.24 million in 2021 and US $3.86 million in 2020 [7]. This viewpoint aims to establish a contextualized model of the IoHT devices’ data that is to be analyzed for assessment of the feasibility of application within AMFA systems. In the *System Architecture of an IoHT Data Model* section, we provide a detailed description of the labels used in the data model proposed for AMFA mutual authentication encryption schemes. The impact of the data classification model is to group the attributes of IoHT environment devices into their respective categories for future research and development purposes. By grouping the devices and their data structures, we aim to provide a foundation to fill the knowledge gap of IoHT systems and their authentication security efforts based on the IoHT literature.

### Literature Review

Authentication in the IoHT is the process of granting access to a user through various steps of multifactor authentication (MFA) to ensure that the user is legitimate. The IoHT is interconnected through numerous applications of communication channels to remotely access and use health data. Remote health care services are more important than ever, not only because of the pandemic but also because of the shift for industries to digitize all their services for better scalability and cost-effective solutions. Table 1 evaluates the literature review for authentication solutions in health care in relation to this paper’s objective toward AMFA. Static authentication is the methodology of an authentication system or solution that configures or has a default setup with no capabilities or intentions to change based on changing environments. Dynamic solutions are those that follow a reactive approach where the authentication methodologies can be changed, or solutions have been provided for restructuring of the authentication system’s capabilities. AMFA seeks to govern or automate the authentication process by considering the security requirements of a system and allowing a proactive approach to security.

**Table 1:** Review of related work based on security features.

Study	Remote authentication solution	Static authentication solution	Dynamic authentication solution	Adaptive authentication solution
Our paper	✓		✓	✓
Azzawi et al [8], 2016	✓	✓		
Baker et al [9], 2017	✓	✓		
Bhatt and Chakraborty [10], 2021	✓	✓		
Kumar et al [11], 2017	✓	✓		
Papaioannou et al [12], 2020		✓		
Scarpato et al [13], 2017		✓		
Sharma and Kalra [14], 2019	✓	✓		

**Figure 1:**
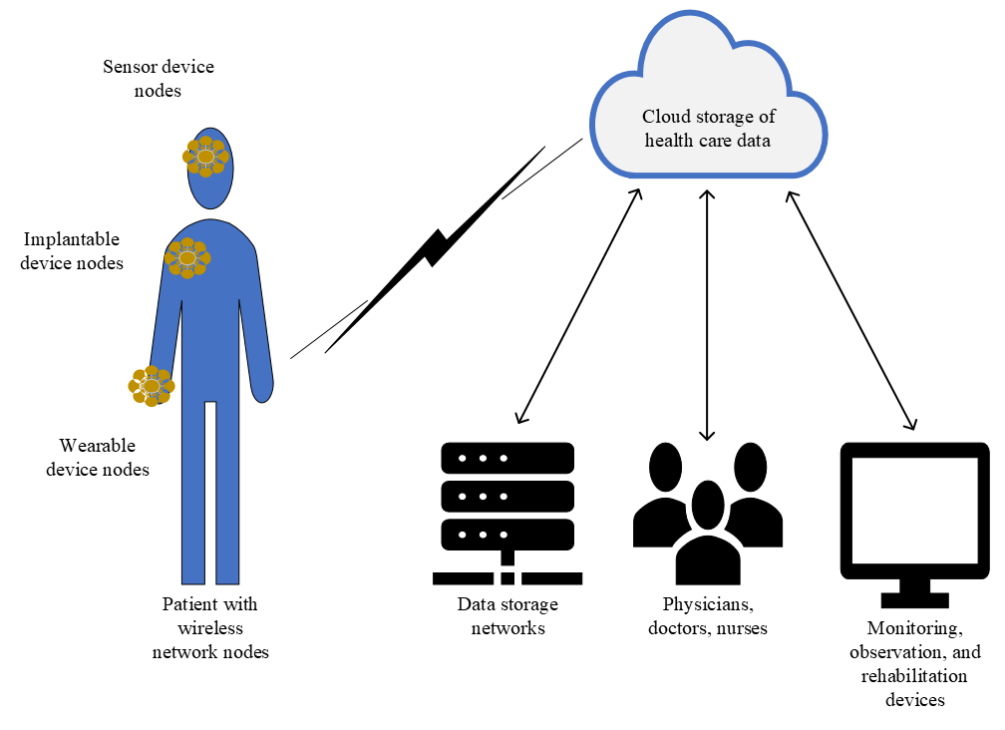
The wireless network of body nodes in an Internet of Health Care Things environment.

Sharma and Kalra [14] proposed a lightweight secure authentication scheme for remote monitoring of patients using an automated validity tool. The protocol in this paper uses timestamps in sensor monitors to mitigate various attacks. However, the proposed solution focuses on an approach to establishing configurations to authentication that is static in nature, which would be unsuitable for an adaptive system that must deploy reactive measures. Azzawi et al [8] proposed an authentication mechanism to reduce the exhaustion of resources in IoT environments. The protocol in this paper uses elliptic curve cryptography to support minimized overhead of resources for encryption. The proposed scheme would be suitable in the IoHT to replace the use of Rivest-Shamir-Adleman encryption to minimize the power constraints on smaller sensor node devices. Papaioannou et al [12] found that the resource constraint on IoHT devices lowers the feasibility of many proposed schemes in the domain of medical authentication schemes. According to their findings, authentication schemes that aimed to implement lower key size solutions would result in lightweight applications that would be well suited to the IoHT to ensure authentication. Scarpato et al [13] proposed a privacy design for IoHT devices, in particular sensors, as their data should not be accessible to all users of the device. However, their principle has not been tested against common IoT attacks, which could be achieved through a user-based AMFA scheme. Kumar et al [11] suggested that biometric authentication has an important role in the IoHT, as lightweight solutions can be designed with high security owing to the strength in biometric solutions. The proposed solution in this paper uses a request and answer algorithm to reduce user interaction throughout the authentication process. However, the scheme promotes centralized systems that would not be secure against various cyberattacks outside their controlled environments, such as in remote authentication scenarios. Baker et al [9] proposed a 4-part model for the IoHT based on a body area network composed of wearable sensors for health care data. The proposed model of this solution is designed to implement various communication technologies such as in Bluetooth or Bluetooth Low Energy (BLE), which are common in small IoT devices owing to their power constraints [9]. However, the proposed encryption of authentication in these technologies is Rivest-Shamir-Adleman encryption, which contains larger key sizes and can constrain IoT devices. The proposed solution also does not protect users from broken authentication should a password system be used, which would be because of poor security posture or overlooking IoT configurations. Bhatt and Chakraborty [10] proposed a smart system for orchestration of health care services to support data sharing in IoHT networks. The research toward this scheme aims to integrate sensor-based devices for real-time environments such as hospitals. This solution requires artificial intelligence for the smart system, which would be beneficial in an AMFA system to govern changing environments of user authentication. As shown in Figure 1, a wireless node network in the human body uses an IoHT environment to generate health care data. Body nodes can use various technologies to facilitate the sensors and monitoring devices for patient care.

## Proposed Methodology

### Overview

IoHT environment security has become a widespread technology of various devices, data, and users to improve health care. To analyze the security requirements of IoHT data classification, it is important to identify the key security features from a cybersecurity perspective. To increase the viability of AMFA in the health care industry, we discovered a shortage of taxonomy for data models that concentrate primarily on IoHT data architecture. In a theoretical framework for a data model that encapsulates the fundamental elements of IoHT data, the purpose of this viewpoint is to identify the major cybersecurity problems. The information is to be applied in ways that are suitable for the COVID-19 pandemic response and current IoHT settings for health care users. A data taxonomy is structured to group data into distinct classes based on shared traits, and the architecture will enhance the comprehension of IoHT data. The taxonomy offers a practical method of categorizing data to demonstrate that it is distinct and without duplication, and these groups contain attributes that serve a purpose in AMFA solutions.

### Why the IoHT?

MFA-IoHT refers to the principles of authentication systems functioning in a health care environment. To improve adaptive systems through machine learning and data models, the MFA-IoHT data must be categorized to consolidate the core attributes that are requirements in IoHT systems. As mentioned in the *Proposed Methodology* section, the four core domains of authentication systems that are crucial to this data taxonomy are as follows: (1) user information, (2) working environments, (3) device information, and (4) use-case settings.

The capabilities of IoHT systems operate on the premise of these 4 domains in relation to AMFA. For the purpose of a data taxonomy, the attributes are categorized within each of these domains and are used to contextualize the data model for a health care environment. Smart eHealth applications such as wearable devices or implantable devices are considered as advanced technologies from an IoT perspective, which rely on privacy and security for their users [15]. These technologies come with unique challenges from a cybersecurity perspective.

### Research Purpose and Contributions

Traditionally, IoT authentication data are handled by default configurations in devices and technologies that are deployed in health care environments owing to their growing number and high demand. This can cause an oversight in security requirements and establish a safe and secure approach to authentication security. This viewpoint contributes toward a better understanding of IoHT data management for AMFA systems by contextualizing security features based on the 4 domains mentioned in the *Why the IoHT?* section. Contextual features are analyzed and discussed such as the attributes of heterogeneous data in the IoHT, that is, user types, device types, use cases, and working environments. The heterogeneous data are then labeled in their respective categories to improve the contextual analysis of AMFA systems in future research and work. To the best of our knowledge, the structured data taxonomy and architecture of IoHT data can help identify and improve the security features of the IoHT by providing a complete overview of the environment.

### Research Criteria and Threats to Validity

It is noted that potential oversight in the selection of research papers and flaws pose the greatest challenges to the validity of this viewpoint, and inconsistencies may exist in the data taxonomy structure. We set the research questions and scopes in advance and manage the selection of research papers based on the 4 methodologies in Table 1 to explore the relevant work of IoHT data management and use in next-generation authentication systems. Reports, journal articles, conference proceedings, and white papers related to remote authentication and user management systems were selected. We also used a variety of search engines to confirm the accuracy of selected content. However, given that this is a nontrivial activity, it is difficult to locate and include all essential research articles in our literature review without excluding any major study efforts. For the validity of this viewpoint, there is a possibility for bias data inputs of heterogeneous IoHT data within the data taxonomy in relation to authentication solutions. We believe that all findings and recommendations were to the best of our knowledge at the time to categorize authentication data.

### Organization of Sections

**Figure 2:**
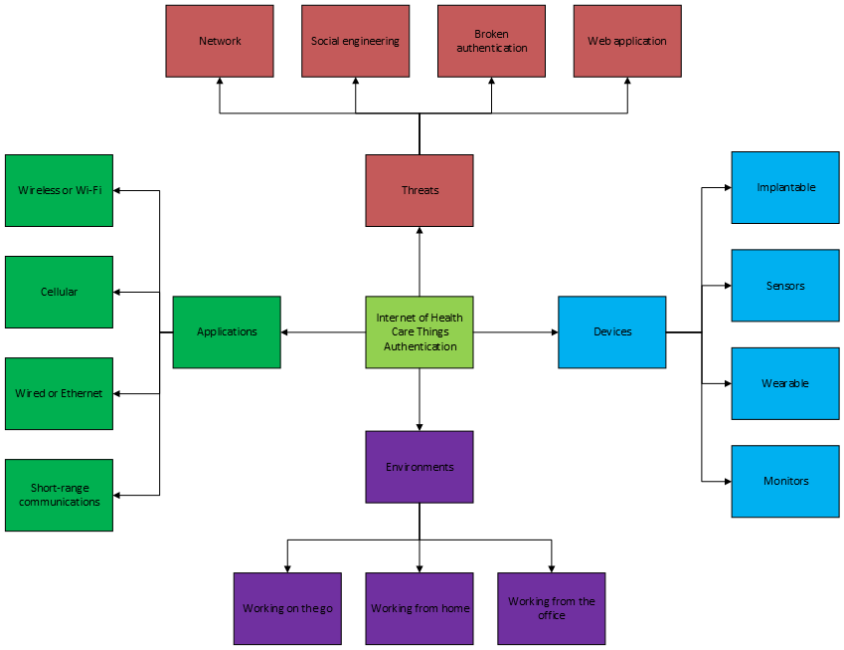
The Internet of Health Care Things authentication layout of the categorized architecture.

The *Research Design and Results* section includes the architecture of the IoHT authentication systems related to health care devices and their respective data. This includes IoHT devices and the communication channels or technologies that are implemented to facilitate their use in health care applications. The *Security Threats in IoHT Authentication* section includes the threats toward authentication in IoT and IoHT networks to establish the security requirements of the proposed contextualized data model for AMFA. The *Theoretical Framework and Discussion of AMFA* section elaborates the theoretical approach toward the contextualized data modality for the AMFA system with respect to each sections’ requirement. Finally, the viewpoint is concluded in the *Conclusions* section. The categorization of the requirements for the data model is provided in Figure 2, showing the main components of the IoHT architecture.

## Research Design and Results

### IoHT Authentication Architecture

Authentication is categorized based on our understanding of the 3 common fields of something you are, something you know, and something you have, allowing us to create MFA combinations of physical traits, knowledge-based traits, and inherence-based traits for security [15]. However, with the requirement for scalability of the growing IoT technologies that emerge, we seek to advance our capabilities with the addition of time and location features in our smart technologies to enhance automation and artificial intelligence–driven responses to handling authenticators [16]. Therefore, in an adaptive solution of the health care data architecture, we must support the diversity and continuity of the authenticators, which are referred to as managed resources that create the elements of authentication [17]. IoHT data can be large in volume, known as big data, and this comes from the vast number of devices that could be used per patient depending on the service being provided; thus, the data become sensitive information as these are often health-related data that are communicated and stored as electronic health records [18]. IoHT devices store, transmit, and communicate data in real time, and these data are often used in critical settings to measure the health of a patient for observation, rehabilitation, and recovery, making protected methodologies of passing the data between legitimate entities a necessity [19].

### IoHT Devices

#### Overview

The architecture discussed in this paper relates to similar systems found in the IoT; in the IoHT, these interconnected systems are contextualized in health care settings, and often, the devices and their users are shared as public resources within a confined practice. Authentication among these devices is important as the initial line of security to establish a safe and secure channel of communication of sensitive information. Textbox 1 shows the categories of devices that we consider in this IoHT authentication architecture. Each category is discussed in detail, and the related papers are assigned in a table format.

**Notation of Internet of Health Care Things devices.**

**Device categories and their notations**

- S_*ID*_: Sensor device [20-25]
- M_*ID*_: Monitoring device [10,23,26-28]
- G_*ID*_: Gateway device [25,29-31]
- W_*ID*_: Wearable device [27,28,32-36]
- I_*ID*_: Implantable device [28,35,37]

#### Sensor Devices

**Figure 3:**
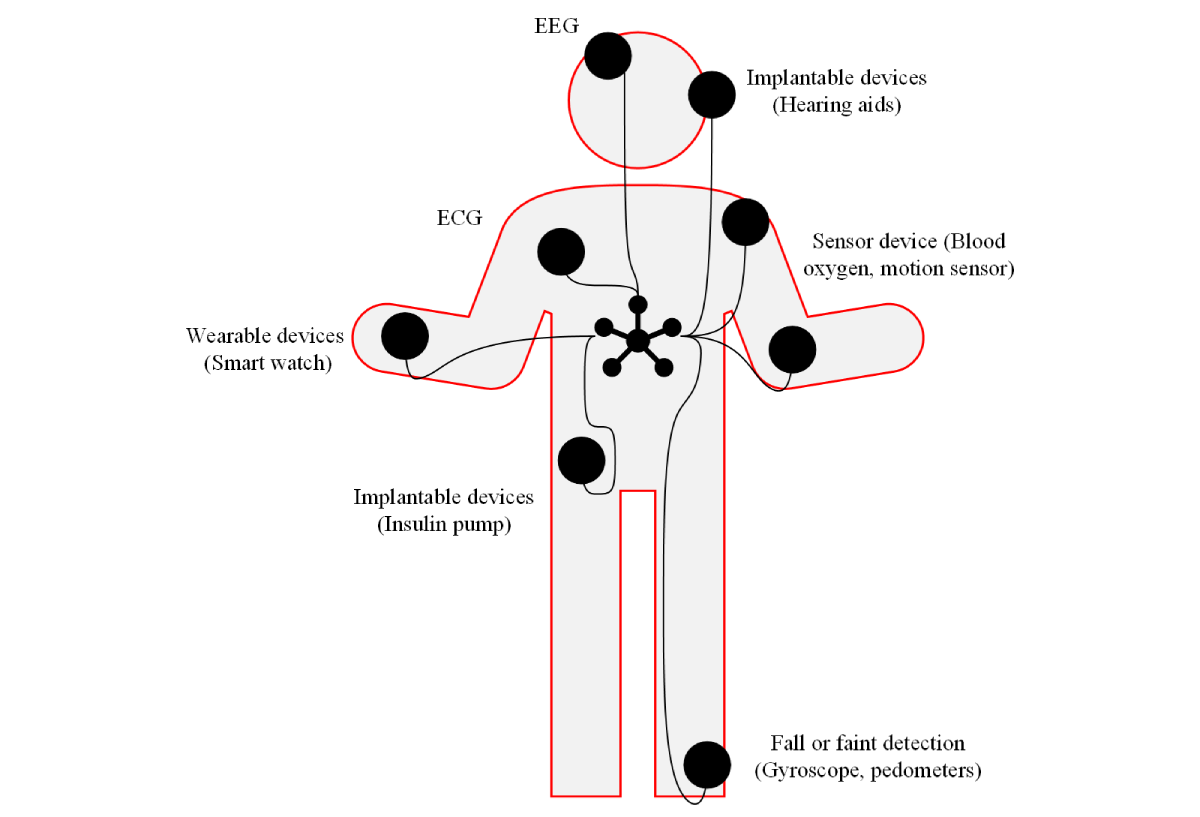
Wireless sensor network of nodes within a human body for health care data generation. ECG: electrocardiogram; EEG: electroencephalogram.

Devices based on sensor node technologies can transmit and communicate data from the sensor device to a monitoring device or storage device either wirelessly or through a wired connection. Wireless sensor devices have emerged in the past decade as physical objects integrated with internet capabilities that provide advanced use of real-time data from the objects *node*, which gives us the terminology of sensor nodes, depicting that IoT devices that can send and receive data [20]. In the context of health care, sensors commonly take the form of wireless medical nodes. These nodes can be implanted, worn, or integrated into patient monitoring systems as a service to oversee various real-time applications. For instance, they can regulate and transmit data such as a patient’s glucose levels or temperature [21]. Medical sensors have improved the quality of life of many IoT devices in health care environments, and they have emerged in the last decade as an industry leader for biomedical data monitoring, known as biosensors, and they can be found in various operating or surgical practices [22]. IoHT applications are greatly improving the use of biometric data reading in health care practices. Biometrics are widely regarded as a robust option for authentication owing to their inherent resistance against replication and forgery. The resilience stems from the challenge of reproducing human traits accurately [25]. Health care monitoring devices have profited from the advancements in sensor technology as collecting personal health data as digital health data and the ability to use cloud resources allow for better use of real-time data in the observation and recovery of patients [23]. Sensor nodes have begun developing 4G and 5G networking capabilities in the health care environments, allowing for allocation of stronger security configurations such as unclonable functions in these sensor nodes to protect against a vast range of threats such as replication attacks, further using existing technologies accessible on most mobile devices to improve scalability [24]. Anwar et al [25] proposed a paradigm for the sensor networks that can be applied to the human body, to read vitals that have expanded as devices become wearable, implantable, and a part of the IoHT architecture. The network is introduced as a Wireless Body Area Network and can be used in health care environments for monitoring various IoHT services for patient care. As shown in Figure 3, sensor nodes in the human body generate biometric data that can be transmitted to health care workers. The different applications of sensors in this figure display examples that can be applied to IoHT networking and then the health care data can be shown in monitoring devices.

#### Monitoring Devices

These devices are used to monitor other devices such as wearable devices, sensors, and implantable technologies, often with their own operating system, and they can use resources such as web applications. Monitoring devices are used for continuous observation and evaluation of patients in a health care environment, meaning that the system is of high importance in critical situations, which could involve monitoring sleep, heart rate, or blood pressure from electrocardiogram (ECG) sensors [10]. Remote monitoring services can also improve the quality of IoT devices in health care settings, as patients can be watched through embedded medical sensors that transmit real-time data, even from home monitors that allow larger volumes of data to be collected over a period to help physicians make better medical evaluations that would not have been possible without constant monitoring at a hospital or clinic in previous times [26]. In addition, in remote health care monitoring, it is possible for these devices to track patient wearable devices such as wristbands with biosensors or implantable devices such as insulin pumps or pacemakers and transmit or store the data to be reviewed later by health care service workers [27]. Monitoring devices also exist as wearable technology that can improve the care of patients through remote or on-site based equipment to collect and monitor their health data, offering mobility and flexibility of patients and the way health care workers can offer their services in working from home or working on-the-go environments that we are seeing more owing to COVID-19 [23]. It is common for real-time monitoring devices to be shared and used by a large volume of different users in a health care environment in on-site environments such as clinics or hospitals, where data are read during an appointment, as opposed to home monitoring devices. Yaqoob et al [28] suggested that monitoring frameworks of IoT applications can be improved with distributed systems that would allow for multiple patients to be able to use a wearable device that would otherwise experience high use of its limited computational resources when treating multiple patients. Monitoring devices can communicate through various technologies such as wirelessly through Bluetooth, Wi-Fi (IPv4 and IPv6), Ethernet wired connections, or even radio frequencies, which are handled by gateway devices that govern the standards and security of communications [27].

#### Gateway Devices

A gateway device is the communication point between devices where data are transmitted, communicated, or stored, and it manages various technologies such as public key infrastructure cryptography, wireless protocols, or Bluetooth communications that make up the networking applications in IoHT architecture. Gateway interfaces include devices that serve as a bridge between the monitoring devices and the data acquisition devices such as wearables and implantable devices, and they address networking protocols to route data in its 3 forms: transmission, storage, and in use [30]. Gateway devices can also be classified as networks of a collection of IoHT devices that interconnect communication application. With the emerging biosensor technologies, wireless body networks are being included as gateway communication nodes within a patient’s body in conjunction with sensors, implants, wearables, and other IoHT devices to store biometric data [25]. 5G networks are being used in the health care sector in combination with IoHT devices to provide fast, reliable, and cost-effective solutions for information communication [31]. Sigwele et al [31] proposed a framework that would use a user’s smartphone as the gateway device between multiple devices to use Bluetooth as a networking application to transmit data and offer an energy-efficient solution to health care services where gateway resources are limited [31]. Cloud computing within health care conveniently accommodates the capabilities of IoHT mobile devices, allowing them to be used further from on-site facilities, such as at home or on the move. This can be achieved through web applications that integrate a user interface for patients to store and access their digital health data while allowing physicians to access the same records from their own office or homes [29].

#### Wearable Medical Devices

The wearable medical devices in the IoHT are responsible for contextualizing real-time data, often from a patient to an electronic record system that can then be further monitored as real-time values or stored values of data for physicians [33]. Wearable medical devices have emerged as a primary approach to handling the large quantities of data shared across devices and systems in health care, with some examples of their use being insulin pumps or other health wellness observation devices that can track and transmit live data in health services [35]. Wearable devices use their integrated sensor technologies to capture patient data such as temperature, heartbeat, oxygen, or glucose, and they transmit their data through these sensor nodes onto monitoring devices, which can then be further measured and analyzed in short- or long-term observations [34]. Wearable devices are an essential tool in health care settings as they advance the flexibility of health care services and can track not only biomedical data but also cognitive or behavioral metrics of a patient such as their mobility- or fitness-related metrics through devices such as smart watches or fitness wrist bands, which can store data over longer periods [32]. Wearable devices have increasing varieties of application in health care, often leaving them vulnerable as their technology’s pursuit robust and lightweight designs that overlook security over functionality [28]. Mo et al [27] proposed a wearable medical device architecture that secures the authentication security of devices through privileged resource management and 2-factor authentication key agreements [27]; this is impactful as the sensitive data found in these IoHT devices are categorized. Wearable devices alongside implantable devices are often limited in their power consumption as they tend to be battery run, and therefore, their security concerns and attack surface must cover offline-based attacks during which the devices are not actively protected by cloud-dependent security services [36].

#### Implantable Medical Devices

Implantable medical devices are often seen as a subcategory of monitoring and sensor devices that analyze patient data, allowing for a contactless approach to persistent observation of vital data and offering a secure service that ensures that a patient’s medical data are kept private, available, and accessible remotely [37]. These devices allow for large data-sharing operations between multiple hardware components such as smartphones, tablets, and display units. From the perspective of authentication technologies, these devices can be configured to allow the use of biometric factors in combination with the device’s physical mechanism to authenticate a user and allow for the transmission, storage, and observations of their personal medical data [35]. The application of wireless biometric sensor nodes in a body network can be established through biometric data within a human to identify and authenticate the user when their health record data are being transmitted via communication channels [25].

### Applications for IoHT Authentication

#### Overview

In this section, we discuss the applications of IoHT devices, that is, the communication technologies of each category. These applications play a crucial role in identifying and mapping the specific attacks that target various devices within a health care authentication environment. The 4 objectives chosen in this section are provided in Textbox 2, demonstrating the classification of the technologies and elaborating on the examples of real-world health care practices of these IoHT devices and their respective data. The examples from Textbox 2 provide a concise overview of patient monitoring, rehabilitation, and observation devices. These examples illustrated the current technological landscape within health care organizations. This section categorizes these devices to establish a framework for defining IoHT elements. This framework addresses the data they manage and the authentication requirements based on user roles, whether they are patients or health care workers. Patients may interact with a device either for personal use or under the guidance of a health care worker. In such cases, the data generated would pertain to the patient and might not necessitate direct access by the patient themselves. Instead, access might be required solely by the health care worker responsible for monitoring or observing the patient’s data, whether within a health care facility or remotely. In addition, devices such as implants, or wearables could also be assigned for rehabilitation objectives.

**Objectives of Internet of Health Care Things devices and data.**

**Remote data communication**

- Radio Frequency Identification [9,38]
- Near-field communication [39-41]
- Wireless networks: Wi-Fi, 4G, 5G, 6G, Bluetooth, and Bluetooth Low Energy [9,28,42]

**Patient monitoring**

- Electrocardiogram monitors, electroencephalogram, electrocardiogram, blood pressure, and blood oxygen [9,23,32,33,43]

**Patient observation**

- Fall or faint detection [44]
- Gyroscope, fitness tracker, and pedometers [45]

**Patient rehabilitation**

- Cloud computing, active assistance, and detection and prevention systems [9,23]

#### Remote Data Communication

In the IoT, there are various technologies that currently exist and are used within the IoHT environments, and remotely communicating data rely on lightweight and robust advances owing to the limitations and constraints of a wireless device to have its own power source [9]. In short-range communications, Radio Frequency Identification (RFID) technology is commonly applied to possession-based factors, such as smart cards or security tags, and is implemented into IoT devices for identity control [38]. Per-tag identification using RFID cards allows for the authentication of a single tag per session by an authenticated user, which can assist in mitigating against the threat of users replicating a user session or hijacking, which will be discussed further in the *IoHT Attacks* section [38]. Near-field communication (NFC) is another short-range localized technology [39] that provides access controls to physical locations and can be used to restrict user access in physical security efforts to ensure that users are authenticated before accessing physical resources. Asymmetric cryptography can be added to tags in addition to one-time passwords to enable remote user identification by keeping the public key inside the device and interacting with the private key inside RFID readers [40]. NFC can also be used in health care monitors to capture the identification of a user or the health care record being used to authenticate the use of the data and then tag or label the entity for tracking as a system [41]. Another set of technologies used in remote data communication are the wireless networks (Wi-Fi), which are very common in most health care industries. These networks use technologies from a wide range of standards such as cellular networks (3G, 4G, and 5G) to Bluetooth for IoHT devices to become interconnected [42]. Bluetooth can be used with IoHT devices for short-range communications with shared key authentication across many IoT devices and is found in most IoT devices in modern times. Bluetooth primarily is designed and used for short-range communication to form smaller networks that are quite flexible, as they support compatibility over various devices, which is favorable in the IoHT [28]. Bluetooth can also be used in BLE modes, which are common in sensor types of IoHT devices as they require a lower amount of power consumption overall, and BLE-capable devices are specifically useful in the design to be fast, cost-effective, and smaller in physical size [28]. Bluetooth technologies can be configured to use different types of encryptions for authentication security and are applied in many real-world environments to connect and share data among large volumes of devices [9].

#### Patient Monitoring

The traditional concept of human monitoring has advanced with the emergence of information and communications technology. These advancements allow complex but lightweight solutions to examine a patient’s health data from a physical perspective to a logical perspective as digital data. To elaborate, this includes remote patient monitoring through IoHT devices as IoT technologies have implemented secure and accurate representations of collecting, storing, and interacting with sensitive health data from various environments such as a physician interacting with their patients in their workplaces, at home, or on the go. Patient monitoring as a field considers the way medical or health care equipment can display or represent the data of a patient such as displaying their vital signs through screens, displays, or other wearable or mobile devices such as ECGs [43]. To contextualize the data, it is crucial to comprehend the heterogeneous data generated by these IoHT devices in health care monitoring. As health care has become more remote owing to the pandemic affecting the world and restraining the health care sector for resources and time, it is important that adaptive authentication can follow suite and allow for better remote authentication options. The IoHT allows monitoring techniques to be applied via take-home or on-the-go devices that use technologies to securely store and transmit their data back to a hospital or personal monitoring device such as a smartphone to display vital health information [9]. An example of a monitoring IoHT device is the ECG monitors, blood monitors, glucose monitors, or other biometric readable devices; these can exist in many forms such as personal devices with displays to show the readings from a small sensor, wearable device, or implantable devices and translate the data into health records of the patient using them [33]. Monitoring requires a high level of security to ensure that only authenticated users can access and interact with the data being recorded by these IoHT devices as an adversary could potentially manipulate the data and misdiagnose a patient or obtain access to prescription drugs that would not otherwise be required [32].

#### Patient Observation

Observation shares a field with patient monitoring when we consider sensor technologies and the nature of IoHT device architecture. The data from a sensor include a patient’s health history, records, or even real-life data such as their vitals that are being transmitted through the IoHT. These sensors are often small communication devices that use various wireless applications of technologies to transmit their data, and their storage space is often small and relies on real-time processing to ensure a reliable flow of power and data in their constrained size limits to fit into common sensor or wearables devices such as smart watches or wearable bands. An example of an observation device is fall or faint detection sensors that were primarily used within health care facilities for a physician to monitor and observe their patients during a time of rehabilitation, but with IoHT advancements, this technology has found itself to be viable remotely, and patients can use these devices from home to alert an authority of their incident [44]. Mobile devices such as fall detection can use wireless technologies to track movement of a patient through wearable or implantable devices using a sensor such as gyroscope [45]. These data can be sent to a gateway or mobile monitoring device; Bluetooth and NFC can be used within health care facilities to monitor patients who are physically present, whereas Wi-Fi can be used for remote monitoring [45]. IoHT devices can also use cloud resources to reduce overhead of data communication on local resources within a health care organization or even on remote resources such as a patient’s gateway devices as they upload and store data from their personal IoHT devices [23].

#### Patient Rehabilitation

Health care rehabilitation has also been improved with IoHT devices and data management in wearable and implantable devices that can provide a service to patients remotely and within a health care facility. Remote consultation through a physician uses the health care data recorded within the devices in conjunction with patient monitoring and observations to provide a smart service to users, which is desirable with the current COVID-19 restrictions in most countries. The monitoring of patients remotely allows artificial intelligence applications to be developed in conjunction with IoHT devices to help patient recovery [10]. IoT-based devices in the health care domain also improve assistance services where wearable or implanted sensors can alert or request resources from health care direct to the patient [23]. Examples of these devices are pushed through the devices to alert health care service providers that a patient requires a service or consulting on demand. Detection and prevention systems are popular in cybersecurity and have application within health care as they allow patient vital information to become reactive [23]. This is useful when applied to biometrics as patient biomedical data such as their blood flow could allow for automated administration of medicines. Cloud-based resources further improve the service of IoHT capabilities by facilitating the management of big data and real-time resource constraints between IoHT devices that are constrained by power consumption [9].

### Security Threats in IoHT Authentication

#### Overview

The main objective of next-generation solutions in this field is to promote a strong posture of security hygiene in the IoT space of health care practices. Security of authentication must be ensured through best practice solutions. To understand how to approach this, we discuss the security requirements of the IoHT devices and their threat and risk landscapes. As shown in Table 2, the threats are categorized, and the security requirements of the IoHT device data taxonomy are discussed.

**Table 2:** Mapping of devices to their corresponding authentication threat types.

IoHT^a^ device	Threat categories			
	Social engineering	Web application	Network	Broken authentication
Sensor device	✓		✓	✓
Monitoring device	✓	✓	✓	✓
Gateway device	✓	✓	✓	✓
Wearable device	✓		✓	✓
Implantable device	✓		✓	✓

^a^IoHT: Internet of Health Care Things.

#### IoHT Threats

##### Overview

The following 3 subsections represent the categories of IoHT threats discussed from the perspective of health care authentication. Threats are categorized as a part of the IoHT taxonomy of data, the model’s threat analysis generates data based on threats to the IoHT within the past decade. Security requirements of the proposed solution were considered from the assessment of the following criteria, social engineering attacks, web application and network authentication threats, and broken authentication attacks.

##### Social Engineering Authentication Threats

Social engineering attacks take advantage of the human factor in a system. In health care, this can be the patients using the services or the physicians interacting with the devices through observation and remediation of their patients’ health [46]. In regard to authenticating IoHT devices, social engineering threats revolve around gathering information concerning a target’s knowledge, possessions, or identity to achieve a position of informational superiority over the target. Social engineering is similar to the reconnaissance phase where the attacker uses their understanding of a system to identify vulnerabilities and get a user or authority with privileges within the system to divulge sensitive information such as credentials or information that would expose the architecture of the authentication system [47]. The most known social engineering attack is a phishing attack, where the attacker sends an email or other type of message to their targets, often containing a malicious link that once opened can infect a system and exfiltrate credentials or other sensitive information [48]. However, attackers could potentially use techniques used in conventional user system attacks to compromise IoHT devices operating autonomously to deliver health services to patients. This could be done in an attempt to escalate ransomware or initial denial of services [49]. Health care data are a high-value target for attackers, who leverage it to exploit patients by establishing their own *authenticity* as legitimate entities during communications. This manipulation frequently results in the coerced divulgence of sensitive information [46].

##### Web Application and Network Authentication Threats

Network-based threats exist through an authentication systems network or through web applications that facilitate the authentication process. These types of attacks are categorized based on web applications that could be vulnerable to injection or forgery of a user or node within an IoHT device that would authenticate malicious attempts from an adversary to attack a system. In web application threats, an adversary can manipulate a weakness or vulnerability found in an application to extract credentials from the user; this can be achieved through interception of the data through a malicious site, injecting malicious code within a vulnerable application and directing the user to it, or even by tampering local resources on the application [50]. Regarding authentication, web applications with inadequate security against access attacks, create a threat surface for attacks with weak or poor management of authentication factors such as using a password [12]. These attacks are especially efficient against IoHT devices, as it is common in IoT devices for security to be set to default parameters that will not detect additional components of a user log-in session such as when or where they logged in from and other security checks to ensure the user is who they claim they are through MFA. Network-based attacks are a category of threats in authentication such as web applications but are not bound to devices or servers, as they can access and manage cloud services, local networks, and other interconnected networks between IoHT devices. Network configurations are designed to be flexible and automated gateways of communication for IoHT devices and their data; however, once an attack is successful, it can quickly scale and increase its volatility through a compromised network [51]. A sybil or replication attack can affect a user identity system, which is often overlooked in terms of security approaches to authentication in IoT networks [12], thus increasing the attack surface of the IoHT through their network communication channels, which must be considered when contextualizing the security of an adaptive model for authenticating users.

##### Broken Authentication Threats

This threat type is based on a subcategory of web applications as its direct platform for staging an attack, but from the perspective of health care authentication, it can also be used through network-based attacks to attack ≥1 accounts in a system to escalate privileges. User management security is often overlooked, and active sessions of a user log-in are not monitored by traditional MFA systems, which will not ensure validity that the user who provided the factors of authentication is the legitimate owner of the user account [52]. Web vulnerabilities exist in poorly configured session management systems that can allow for an adversary to manipulate a session, even copy it, or forge a malicious session that imitates the legitimate one to avoid detection from users and security authorities of the system during an attack [52]. This type of threat can establish the basis of the previously mentioned threat types as a starting point for an adversary to further escalate their attacks within a system once they have obtained credentials relevant to the network [53]. Offline attacks are another angle that an adversary can take toward broken authentication of users. Password authentication poses a great threat in this category because there are many applications for staging an attack on an authentication factor such as a smart card, where an adversary can replicate or clone a device and begin to attempt every possible password combination until they are successful without ever alerting the actual system [54].

#### IoHT Attacks

##### Overview

**Figure 4:**
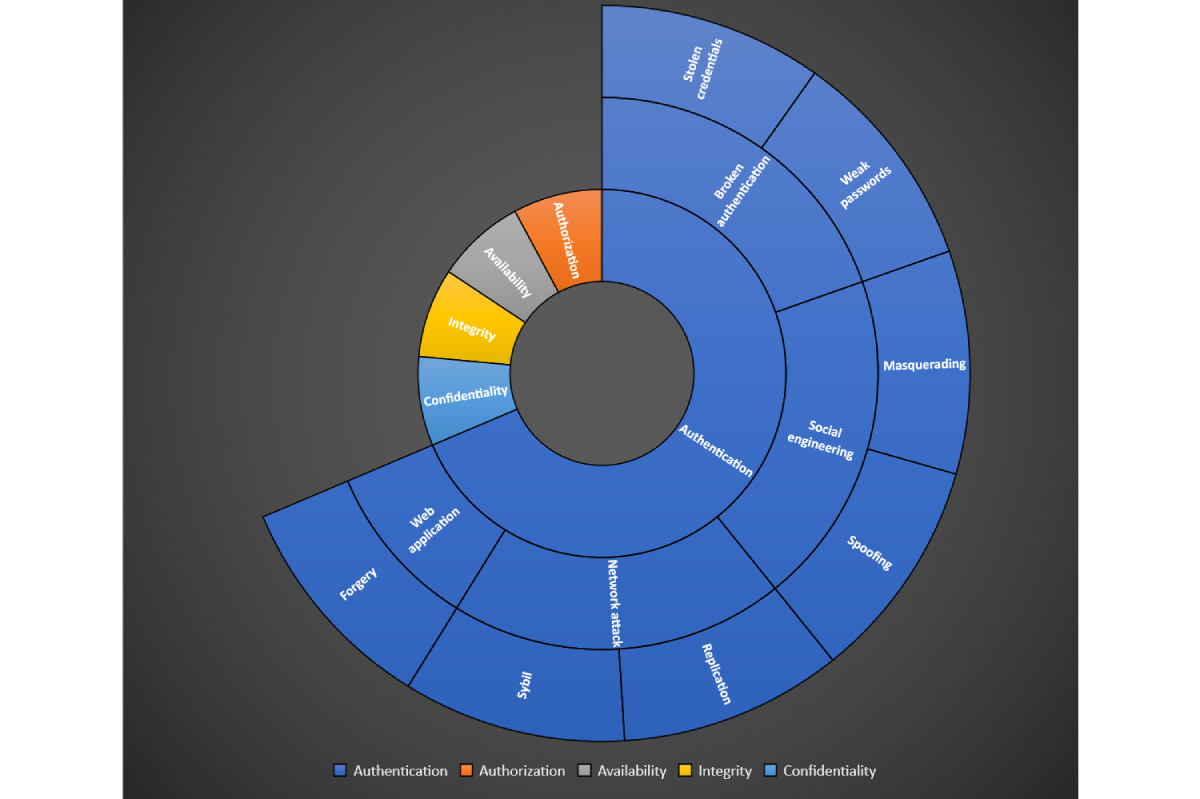
Attack chart for the authentication category in the field of computer science.

In this section, we discuss the attacks that IoHT authentication architecture faces in a health care context. Figure 4 shows the 5 major components of security research: confidentiality, integrity, availability, authorization, and authentication. The scope of these attacks lies within authentication factors and their corresponding data management within the health care context. In this section, we define the threats and attacks to IoHT data and devices for categorization. In Table 3, we summarize the attack types as they are mapped out to their relevant threat categories regarding IoHT authentication. It is important to note that these are the in-scope objectives of this paper, but many threats arise and challenge the health care sector as more data become digitalized.

**Table 3:** Threat categories of different authentication attack types.

Threat tag	Threat category	Attack type
A_1_	Social engineering	Spoofing or masquerading attacks [19,28,55]
A_2_	Web application	Forgery attacks [12,56-58]
A_3-1_	Network	Replication attacks [12,14,59]
A_3-2_	Network	Sybil attacks [12,20,60,61]
A_4-1_	Broken authentication	Weak passwords [15]
A_4-2_	Broken authentication	Stolen credentials [54,62]
A_4-3_	Broken authentication	Access attacks [63]

##### Masquerading or Spoofing Attacks

Masquerading or spoofing attacks in the context of authentication fall under the social engineering threat category. Masquerade attacks against IoT devices can affect both wired and wireless technologies in a health care setting and can be used to remotely impact a user’s authentication process and affect the privacy of a system [55]. Spoofing a user’s authentication involves the manipulation of a legitimate user through vulnerabilities in the IoHT device or application being used, such as through redirection to a malicious source of a session that impersonates a legitimate channel of communication or site [19]. Once an attacker has spoofed the legitimate user, they can manipulate credentials or other sensitive data that can be used to leverage their way through the authentication system by elevating their node, and it is often achieved by masquerading, as the users they have affected to further exfiltrate sensitive data or spread their attack capabilities from within the system [19]. Adversaries can spoof medical practices to intercept patient medical data transmitted or stored in IoHT devices in combination with other attacks and vulnerabilities, including wearable and implantable devices [28].

##### Forgery Attacks

Forgery attacks are often cross-site requests or scripting attacks found in web browser applications and target authentication or authenticated users. This attack forces interactions with exploited services that can lead to exposed credentials or unauthorized actions through remote user manipulation [56]. Forgery attacks are similar to masquerading attacks and involve a 2-part process. The first part involves creating an identity within the system to pose as a legitimate entity; an adversary then uses this deceptive identity to entice genuine users into engaging with their forged attack [12]. In the second part of this attack, the adversary can fabricate IoHT devices and other nodes to steal existing authentications from legitimate users and maintain their leverage within a system [12]. Remote user authentication suffers from having lower security postures in bad practices such as the handling of passwords in IoHT devices where credentials are not hashed with cryptographic measures to obscure plaintext stored information [58]. It is easy for an adversary to fake certificates on public key exchanges, which can extract the private key of a legitimate user, during interaction with a client, website, or service from a fraudulent source that an adversary has prepared [57].

##### Replication Attacks

In the context of health care authentication, a replication attack can involve the cloning or replication of a device that often is linked to a singular device or sensor via a unique identifier such as a MAC address [12]. An adversary can take advantage of devices that do not provide authentication security options for detecting log-ins from a given location based on where the device should be situated in its given work environment [12]. Often a sybil attack occurs on IoT devices as they use wireless technology to communicate and store the data within the device through the sensor nodes. Wireless sensor technology networks are vulnerable to node replication and sybil attacks because many of the components that make up a node are left defenseless and often on their default configurations out of the box, making their attempts at security often trivial to attackers with knowledge of the device [59]. Wireless sensor devices are often lightweight technologies that communicate closely with other IoHT devices to form a large network of monitorable sensitive data in which an adversary will manipulate interception of communicating applications to control these nodes and where they send their data [14].

##### Sybil Attacks

Sybil attacks share some properties with replication attacks, except that the adversary can extend a hijacked or replicated node to gain influence within the network through other created identities or nodes [12]. This type of attack affects an identity network by gaining a large portion of nodes within an IoT device and overcoming a “reputation system,” which refers to identity structures where poor security has been implemented by giving users rights to certain actions within a system that they would not normally have without many identities [20]. In a remote IoHT device where mobile networks are being used, the adversary can manipulate local resources within the system. They can exploit their *majority* of identities to influence decisions that would be unobtainable for a single user [60]. By publishing multiple malicious nodes of the adversary’s identity, it is possible for the attacker to route messages or other types of sensitive information within the IoHT device into their possession for manipulation or exfiltration [61].

##### Access Attacks, Weak Passwords, and Stolen Credentials

Broken authentication is a broader category of challenges and attacks such as brute force, weak passwords, stolen credentials, and credential stuffing. This category shares similar principles among each subcategory of attack based on weak security measures and can be related to social engineering approaches. Access attacks are an attempt by an adversary to access a legitimate user’s account through manipulation, intrusion, or forceful measures, often using third-party information where a data breach or use of a reused password that has been exposed in the past has been used again [15]. Weak passwords can be obtained through brute forcing, dictionary attacks, or by using rainbow tables. Adversaries exploit system vulnerabilities to acquire passwords, attempting every conceivable combination based on their findings related to the user account [62]. According to Bošnjak et al [62], weak passwords are still being used presently despite the vast range of research and statistics that point to the use of passwords being one of the weakest approaches to authentication security, and they claim that a modern graphics processing unit can crack >95% of passwords in only a few days. Botnets are another way that an adversary can perform access attacks on authentication systems as they use a large volume of bots to perform password guessing or password cracking attacks on large identity systems such as a health care identity database, attempting to escalate their privileges in the network through higher-value users [63]. Offline password guessing is mainly a weakness in wireless sensor nodes found in IoHT devices because of the lack of an MFA security feature configuration. An example of a threat to an authentication factor’s security features is within single-factor authentication security. A device such as a smart card is vulnerable to tampering if a weak or stolen password can bypass the single layer of security during authentication, thereby exposing the user’s data [54].

## Theoretical Framework and Discussion of AMFA

### Overview

Throughout this paper, we have discussed the applications of AMFA in IoHT domains based on authentication security requirements. In addition, we have discussed AMFA in the context of health care environments and evaluated the feasibility of an improved AMFA model that can address security concerns over IoHT methodologies. In the design of this data taxonomy, we consolidated the 4 domains of MFA systems: user information, working environments, device information, and use-case settings.

In the following sections, we discuss the foundation of the data taxonomy proposed as a solution for AMFA data management. We elaborate on the categorization of the attributes that are regarded for an AMFA systems in relation to the 4 domains of the data taxonomy. On the basis of our findings, the relationship between MFA attributes and IoHT data is summarized in a data model. The resulting taxonomy of the AMFA-IoHT data consolidates the emerging disciplines of AMFA research fields to improve security requirements in adaptive authentication systems. These data can be used to improve the scalability of existing MFA solutions in the current health care environment, and the adaptability of authentication systems can be improved with privacy and security.

### System Architecture of an IoHT Data Model

#### Overview

**Figure 5:**
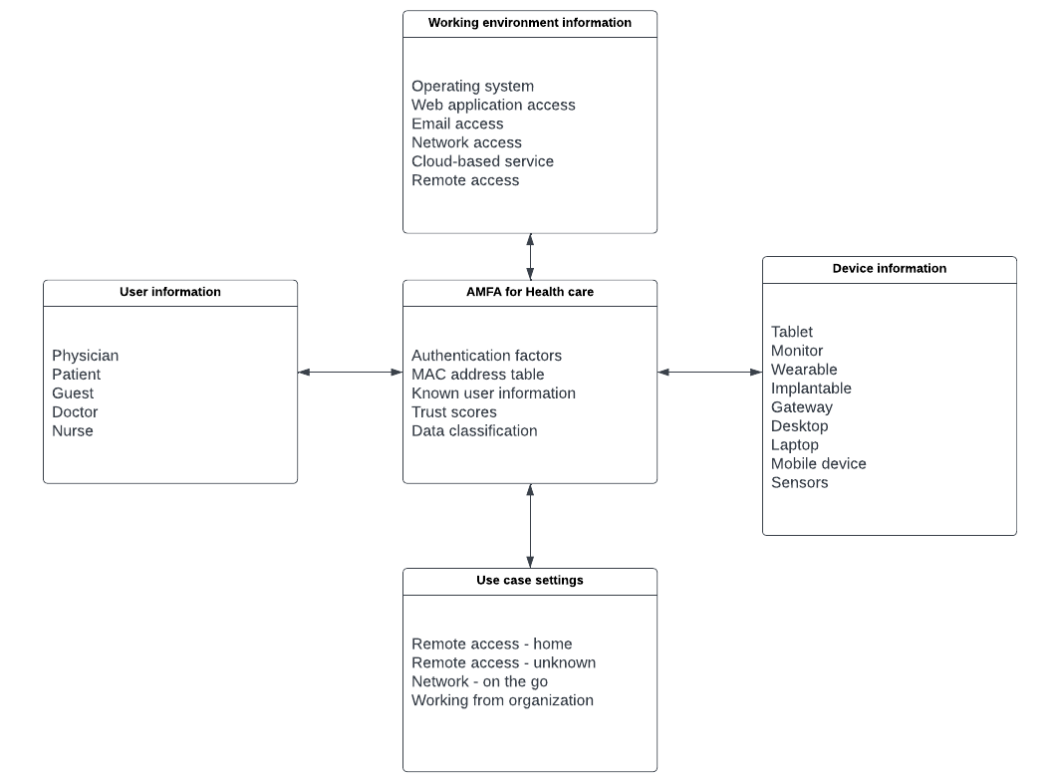
Elements of heterogeneous data in the Internet of Health Care Things. AMFA: adaptive multifactor authentication.

The attributes discussed in this section can be categorized, as shown in Figure 5, to contextualize the data model within their respective categories, which combines user types with data types and device types. The security requirements of an AMFA system in the IoHT must ensure reliability, scalability, and lightweight design to reduce constraints on resources, especially where smaller technologies such as sensors are used. These attributes form a taxonomy of IoHT data structures categorized by devices, users, and the corresponding environment of use. This classification also takes into account the potential threats that these entities might encounter. We generate these attributes to be used in the autonomy of security approaches to an AMFA solution that benefits users such as the older adults who could be overwhelmed with the authentication options they are presented with. In addition, we aim to minimize the expenses and setup complexity associated with automating an AMFA solution. This is particularly important as human errors and negligence tend to arise when transitioning toward enhanced security measures that moves beyond reliance on passwords.

Building upon the insights from our analysis in Figure 5, the elements selected for the IoHT architecture contribute toward an AMFA approach to security requirements. The threats that challenge IoHT data are based on data breaches and attacks that affect user nodes through creation or manipulation. Health care requirements against these challenges persist even after the current situation of the COVID-19 pandemic. The leading cause of poor security approaches to these challenges is poor or weak authentication methods, such as the use of passwords in user-based systems. Our solution provides a data model for the automation of IoHT architecture to reduce user interaction with the selection criteria of authentication factors. The proposed system was designed to adapt on the changing features of the IoHT environment.

#### Use-Case Settings

The criteria for the 3 use cases are used within the conceptual model for remote access to health care data, as shown in Table 4. In Table 4, the decision-making criteria for the use-case settings are used to address COVID-19 constraints on physicians and patients that would be impacted when authenticating remotely. Each use-case is labeled with (C_#_) to be used in further computational assessments of the proposed solution. The environments are described based on theoretical data gathered from our research on the security requirements and nature of IoHT devices and their respective data. Our proposed solution is suitable for the current restrictions and constraints, which promote authentication toward a passwordless approach to adaptive security systems.

**Table 4:** Table of use cases for health care environment labels.

Label	Name	Description
C_1_	Remote access authentication	Whether a device is being accessed remotely outside of the health care fixed network and beyond private firewalls or session security on business networks; this could be outside of business hours and includes free Wi-Fi networks from public access networks
C_2_	Working from home authentication	A device being accessed from a home network either through a personal network or a business portal that is regulated
C_3_	Working from within the organization authentication	Fixed networks being used through organization networks, portals, servers, or cloud resources with security configurations within business hours or from within the organization’s geolocation

#### Data Classification Criteria

As shown in Table 5, the classification of the data for the proposed model can be labeled as (L_#_) depending on the findings of the research once it commences. Each level can be determined through an analysis of the related studies to design a comparative study of the sensitivity of the data contained within IoHT devices. As discussed in the *Literature Review* section, it is important for there to be a distinction of data classification to improve security features. The requirements of the data model can be expanded with each level applied to the data in these IoHT devices when research and development of real-world solutions are applied.

**Table 5:** Table of data sensitivity labels.

Label	Name	Description
L_1_	Low sensitivity	Low sensitivity depending on the nature of the data on the device such as name of the patient or workers, home address, the preferred or most recently used health care services, email address, and contact details
L_2_	Medium sensitivity	A medium sensitivity rating for IoHT^a^ data that include identifying information such as government number, income-related information, health care information, date of birth, and other identifiers of the persons
L_3_	High sensitivity	High sensitivity data are labeled for IoHT data that include personal information such as health conditions, health history, prescription history, medical records, payments for health care such as Medicare in Australia, biometric data, or media files regarding the patient or workers (photo identification, user credentials, etc)

^a^IoHT: Internet of Health Care Things.

#### User Information Criteria

As shown in Table 6, the user information labels are set based on the findings of the user types we have established within the paper for health care environments, as both workers and patients of health care services. The label for the proposed model can be defined as (U_#_). The user information is an important requirement of the data model to ensure data privacy in conjunction with data sensitivity. This allows future research and development of IoHT devices such as sensors as a requirement of ensuring privacy over shared devices that handle many users’ digital health data.

**Table 6:** User information labels.

Label	Name	Description
U_1_	Health care employee	This includes doctors, nurses, physicians, or any other form of certified user that has a legitimate account with the health care organization.
U_2_	Patient	This includes any person or entity tied to an account receiving a health care service either remotely or from within a health care organization using IoHT^a^ devices.
U_3_	Guests	This includes any type of user who is accessing limited access from an account to display IoHT data with constraints on their access.

^a^IoHT: Internet of Health Care Things.

### Limitations of the Data Model Taxonomy

**Figure 6:**
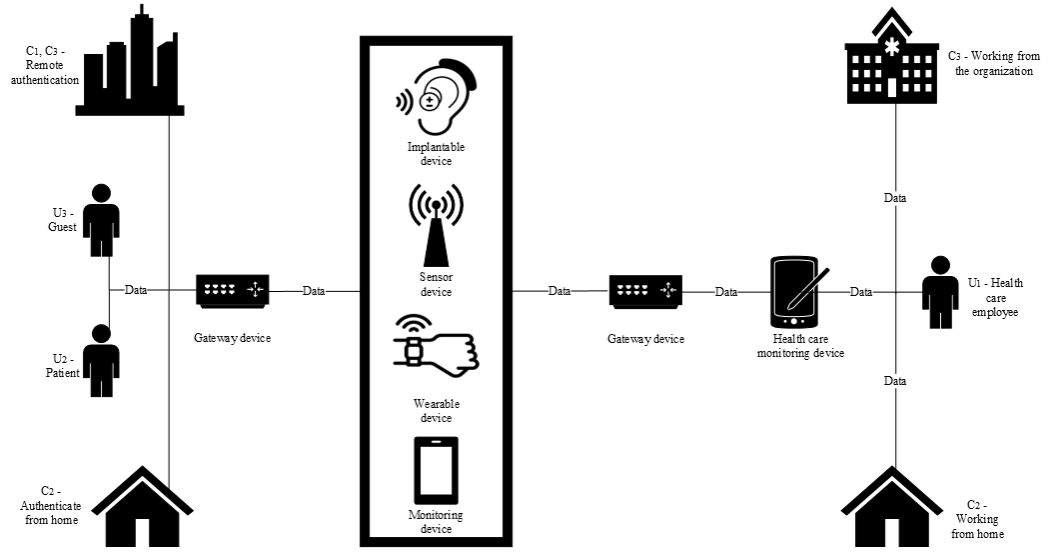
A conceptual structure of data in motion in an Internet of Health Care Things environment. C1: remote access authentication; C2: Working from home authentication; C3: Working from within the organization authentication; U1: health care employee; U2: patient; U3: guest.

The IoHT network architecture comprises numerous security domains, including sensors, monitoring equipment, data management, and dispensary systems [12]. The attack surface of IoHT networks is large and vulnerable to a variety of cyber threats internally and externally [12] as discussed in our threat categories. The purpose of this data model is to establish a categorization for authentication and the security threats to IoHT networks. Categorization is essential for improving security features based on the following requirements: the “entities” in a conceptual model are components of the overall system or the essential resources and services that make the entire system functional. The IoHT is a large and continuously evolving industry of products that facilitate in digitalizing services, networks, and information systems. Identifying the key objectives with a focus on AMFA, based on the reviewed literature, contributes toward adaptive factor selection. Authentication factors are categorized into the 3 classifications: something you are, something you know, and something you have. These categories cover the known and most used factors in authentication security approaches and do not need to be improved because they cover the parameters of adaptive security appropriately. To ensure adaptive security, our research adopts the approach of a passwordless MFA security system. As shown in Figure 6, data communicate between sensors and monitoring devices in a health care environment and contain crucial information to be transmitted in real time. This is classified as sensitive data important for authentication and a classification scheme. The structured data ensure that when health care workers begin authentication, it is safe and time efficient, meaning there is less unnecessary complexity for security features. This supports a better understanding of the devices that are affected (ie, sensors, monitors, and other remote devices), allowing for appropriate classification based on authentication factors chosen. The outcome of this phase creates an algorithmic metric that gives resources a weighting to categorize them as low, medium, or high sensitivity.

### Adapting Traditional MFA to IoHT Authentication

**Figure 7:**
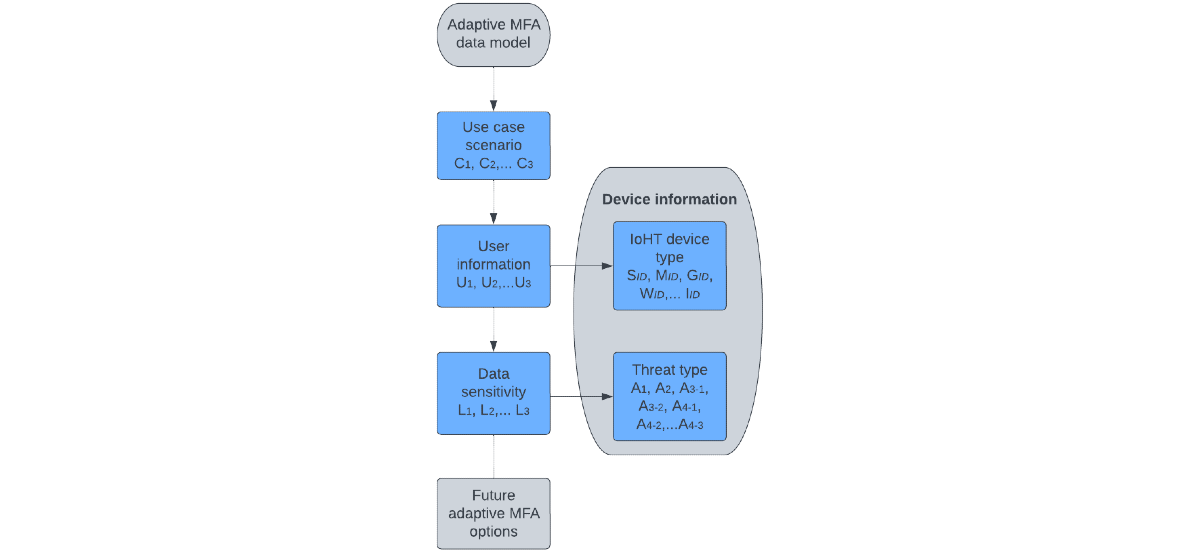
Contextualized Internet of Health Care Things (IoHT) architecture toward an adaptive multifactor authentication (MFA) model. A1: spoofing or masquerading attacks from the social engineering category; A2: forgery attacks from the web application category; A3-1: replication attacks from the network category; A3-2: sybil attacks from the network category; A4-1: weak password attacks from the Broken Authentication category, A4-2: stolen credential attacks from the Broken Authentication category; A4-3: access attacks from the Broken Authentication category; C1: remote access authentication; C2: Working from home authentication; C3: Working from within the organization authentication; GID: gateway device; IID: implantable device; L1: low sensitivity; L2: medium sensitivity; L3: high sensitivity; MID: monitoring device; SID: sensor device; U1: health care employee; U2: patient; U3: guest; WID: wearable device.

As discussed in the previous sections, AMFA uses entities to improve the scalability and allocation of the administrative system that determines which factors to present to a user based on their given environment. The current solution for IoHT authentication is the traditional public key infrastructure structure, which uses certificate-based methodologies to store, verify, and monitor permissions or trust that a user has within a system or network [64]. Nag and Dasgupta [65] proposed a continuous MFA solution for identification of users through virtualization of resources that could distribute user authentication and allow controlled access through an established server access network. The issue we find in these solutions comes from the possibility of forgery attacks, masquerading attacks, or replication attacks that would allow a perpetrator to obtain complete control of the AMFA system, thus making previous approaches infeasible to modern IoHT networks. A recent survey on adaptive authentication claims that the challenge with implementing an adaptive security factor into integrated systems is the inability to reuse or introduce new authentication factors as they arise in emerging trends of passwordless approaches to MFA [17]. To address this, we label the devices in the context of health care distributed networks and categorize them into different groups or modalities for the data classification proposed model, creating a platform of which different authentication factors or devices can be introduced into the developing algorithms that are expected to be developed from these findings and discussions. Furthermore, this will enable a push forward for adaptive security efforts by providing a novel direction in building an open platform model that can adapt to the emerging environments in the IoHT as modern technologies and conditions develop. AMFA can derive advantages from a contextually structured data model meticulously crafted for the health care sector. This involves designing and implementing authentication factors that align with the unique requirements of health care organizations and the IoHT devices’ perspective on data. Moreover, it entails determining how these methods can be effectively applied to users based on temporal and environmental factors. The objective is to balance the security needs of both the users and the sensitivity of the data being stored, transmitted, or used, and when this occurs, as shown in Figure 7, the labels created within this paper can be used to conceptualize a future approach to AMFA options.

### Conclusions

We have designed various approaches toward labeling a complete data model taxonomy for IoHT networks. This paper demonstrates our categorization of devices and their respective data in correspondence with health care objectives and services such as patient monitoring, observation, and recovery. Health care as a service through the IoHT has vastly improved the capabilities of the industry and how patients can access the services from home or on the go as social distancing mandates in many countries around the world are still in place to help reduce outbreaks of new variants of COVID-19 [4]. Information and communication technologies through wireless networks such as existing 4G and 5G networks have facilitated the remote access or working from home trends in recent years as organizations have been forced to move most of their services through digital communication to mitigate the threat of the virus and the social impacts to their workforce [66]. The IoHT domain is vulnerable to many cyber threats; therefore, IoHT devices must be designed with these security requirements to ensure reliable authentication practices. By reviewing the literature in this paper, we have established a theoretical framework toward AMFA in an IoHT network addressing the privacy concerns of users and the data management of these devices in a health care environment.

**Figure 8:**
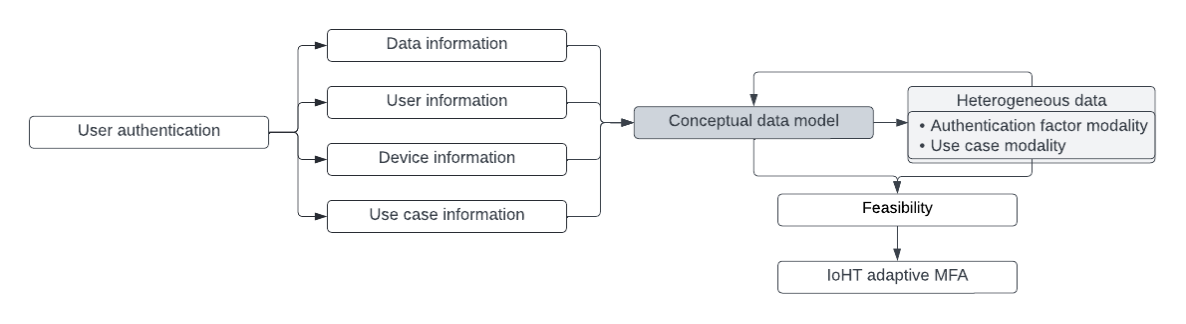
Adaptive multifactor authentication (MFA) model flowchart with the current stage of research. IoHT: Internet of Health Care Things.

With the main objective of mapping out the IoHT architecture to include devices and their data with respect to the technologies used to digitalize communications in an interconnected IoT network, we can see that further research will be required to help improve the health care industry toward an AMFA model, where a user can be authenticated without reliance of weak authentication and become passwordless. The data taxonomy of the 4 domains we have discussed as security requirements of AMFA-IoHT data promote the privacy and security features for authentication systems in health care. An adaptive authentication system’s feasibility consolidates the 4 domains as primary principles for user authentication schemes and the cyber threats that they face. As shown in Figure 8 and Table 3, a conceptual data model can be used in combination with authentication factors and consideration of a user’s working environment toward the feasibility of an AMFA-IoHT model.

Heterogeneous data collected from an improved adaptive authentication factor selection criterion in our future research development can be applied to the flowchart depicted in Figure 7 to help improve our understanding of IoHT devices and data information and how cyberattacks impact their deployment ability within a health care enterprise. Numerous MFA solutions are appropriate for selection in the AMFA system, which extends the scope and timeline of the security requirements. Therefore, to mitigate scope creep in the decision-making criteria for authentication factors, the following future research and development criteria will be established:

1. Factors from each category of knowledge, possession, and inherence will be selected based on their feasibility in an IoHT environment.
2. For the AMFA as a service, the factor combinations must be flexible while maintaining practicality.
3. The factor list can be expanded or contracted based on reiteration of the literature and findings in the contextualized data model during informal analysis of the data classification attributes in the existing models.

## Abbreviations

AMFA: adaptive multifactor authentication
BLE: Bluetooth Low Energy
ECG: electrocardiogram
IoHT: Internet of Health Care Things
IoT: Internet of Things
MFA: multifactor authentication
NFC: near-field communication
RFID: Radio Frequency Identification

## Data Availability

All data generated or analyzed during this viewpoint are included in this manuscript.

## References

[1] Javaid M, Khan IH (2021). Internet of Things (IoT) enabled healthcare helps to take the challenges of COVID-19 Pandemic. J Oral Biol Craniofac Res.

[2] Bradley C, El-Tawab S, Heydari MH (2018). Security analysis of an IoT system used for indoor localization in healthcare facilities. Proceedings of the Systems and Information Engineering Design Symposium (SIEDS).

[3] Adetunji CO, Olaniyan OT, Adeyomoye O, Dare A, Adeniyi MJ, Alex E, Rebezov M, Petukhova E, Shariati MA, Pani SK, Dash S, dos Santos WP, Chan Bukhari SA, Flammini F (2022). Internet of Health Things (IoHT) for COVID-19. Assessing COVID-19 and Other Pandemics and Epidemics using Computational Modelling and Data Analysis.

[4] Fagroud FZ, Toumi H, Ben Lahmar EH, Talhaoui MA, Achtaich K, Filali SE (2021). Impact of IoT devices in e-health: a review on IoT in the context of COVID-19 and its variants. Procedia Comput Sci.

[5] Murugan S, Vijayakumar K, Sivakumar V, Manikandan R, Kumar A, Saikumar K, Anandan R, Suseendran G, Chatterjee P, Jhanjhi NZ, Ghosh U (2022). Impact of Internet of Health Things (IoHT) on COVID-19 disease detection and its treatment using single hidden layer feed forward neural networks (SIFN). How COVID-19 is Accelerating the Digital Revolution.

[6] (2021). IBM report: cost of a data breach hits record high during pandemic. IBM Newsroom.

[7] Cost of a data breach report 2022. IBM Corp.

[8] Azzawi MA, Hassan R, Bakar KA (2016). A review on Internet of Things (IoT) in healthcare. Int J Appl Eng Res.

[9] Baker SB, Xiang W, Atkinson I (2017). Internet of things for smart healthcare: technologies, challenges, and opportunities. IEEE Access.

[10] Bhatt V, Chakraborty S (2021). Real-time healthcare monitoring using smart systems: a step towards healthcare service orchestration smart systems for futuristic healthcare. Proceedings of the International Conference on Artificial Intelligence and Smart Systems (ICAIS).

[11] Kumar T, Braeken A, Liyanage M, Ylianttila M (2017). Identity privacy preserving biometric based authentication scheme for Naked healthcare environment. Proceedings of the IEEE International Conference on Communications (ICC).

[12] Papaioannou M, Karageorgou M, Mantas G, Sucasas V, Essop I, Rodriguez J, Lymberopoulos D (2020). A survey on security threats and countermeasures in Internet of Medical Things (IoMT). Trans Emerging Telecommun Technol.

[13] Scarpato N, Pieroni A, Di Nunzio L, Fallucchi F (2017). E-health-IoT universe: a review. Int J Adv Sci Eng Inf Technol.

[14] Sharma G, Kalra S (2018). A lightweight user authentication scheme for cloud-IoT based healthcare services. Iran J Sci Technol Trans Electr Eng.

[15] Ometov A, Petrov V, Bezzateev S, Andreev S, Koucheryavy Y, Gerla M (2019). Challenges of multi-factor authentication for securing advanced IoT applications. IEEE Network.

[16] Ometov A, Bezzateev S, Mäkitalo N, Andreev S, Mikkonen T, Koucheryavy Y (2018). Multi-factor authentication: a survey. Cryptography.

[17] Arias-Cabarcos P, Krupitzer C, Becker C (2019). A survey on adaptive authentication. ACM Comput Surv.

[18] Thara DK, Premasudha BG, Ram VR, Suma R (2016). Impact of big data in healthcare: a survey. Proceedings of the 2nd International Conference on Contemporary Computing and Informatics (IC3I).

[19] Mamdouh M, Awad AI, Khalaf AA, Hamed HF (2021). Authentication and identity management of IoHT devices: achievements, challenges, and future directions. Comput Secur.

[20] Arshad A, Mohd Hanapi Z, Subramaniam S, Latip R (2021). A survey of Sybil attack countermeasures in IoT-based wireless sensor networks. PeerJ Comput Sci.

[21] Kirsal Ever Y (2019). Secure-anonymous user authentication scheme for e-healthcare application using wireless medical sensor networks. IEEE Syst J.

[22] Javaid S, Zeadally S, Fahim H, He B (2022). Medical sensors and their integration in wireless body area networks for pervasive healthcare delivery: a review. IEEE Sensors J.

[23] Mora H, Gil D, Terol RM, Azorín J, Szymanski J (2017). An IoT-based computational framework for healthcare monitoring in mobile environments. Sensors (Basel).

[24] Yu S, Park Y (2022). A robust authentication protocol for wireless medical sensor networks using blockchain and physically unclonable functions. IEEE Internet Things J.

[25] Anwar M, Abdullah AH, Qureshi KN, Majid AH (2017). Wireless body area networks for healthcare applications: an overview. Telkomnika.

[26] Kintzlinger M, Nissim N (2019). Keep an eye on your personal belongings! The security of personal medical devices and their ecosystems. J Biomed Inform.

[27] Mo J, Shen W, Pan W (2020). An improved anonymous authentication protocol for wearable health monitoring systems. Wirel Commun Mob Comput.

[28] Yaqoob T, Abbas H, Atiquzzaman M (2019). Security vulnerabilities, attacks, countermeasures, and regulations of networked medical devices—a review. IEEE Commun Surv Tutor.

[29] Jemal H, Kechaou Z, Ayed MB, Alimi AM (2015). Cloud computing and mobile devices based system for healthcare application. Proceedings of the IEEE International Symposium on Technology and Society (ISTAS).

[30] Pradhan B, Bhattacharyya S, Pal K (2021). IoT-based applications in healthcare devices. J Healthc Eng.

[31] Sigwele T, Hu YF, Ali M, Hou J, Susanto M, Fitriawan H (2018). Intelligent and energy efficient mobile smartphone gateway for healthcare smart devices based on 5G. Proceedings of the IEEE Global Communications Conference (GLOBECOM).

[32] Albesher AA (2019). Iot in health-care: recent advances in the development of smart cyber-physical ubiquitous environments. Int J Comput Sci Netw Secur.

[33] Fernandez F, Pallis G (2014). Opportunities and challenges of the Internet of Things for healthcare. Proceedings of the 4th International Conference on Wireless Mobile Communication and Healthcare - "Transforming healthcare through innovations in mobile and wireless technologies".

[34] Haghi M, Thurow K, Stoll R (2017). Wearable devices in medical Internet of Things: scientific research and commercially available devices. Healthc Inform Res.

[35] Hudson F, Clark C (2018). Wearables and medical interoperability: the evolving frontier. Computer.

[36] Tawalbeh L, Muheidat F, Tawalbeh M, Quwaider M (2020). IoT privacy and security: challenges and solutions. Appl Sci.

[37] Wu L, Du X, Guizani M, Mohamed A (2017). Access control schemes for implantable medical devices: a survey. IEEE Internet Things J.

[38] Kang J, Fan K, Zhang K, Cheng X, Li H, Yang Y (2021). An ultra light weight and secure RFID batch authentication scheme for IoMT. Comput Commun.

[39] Al-Saedi SB, Azim MM (2017). Radio Frequency Near Communication (RFNC) technology: an integrated RFID-NFC system for objects' localization. PrOceedings of the 9th IEEE-GCC Conference and Exhibition (GCCCE).

[40] Newaz AI, Sikder AK, Rahman MA, Uluagac AS (2021). A survey on security and privacy issues in modern healthcare systems: attacks and defenses. ACM Trans Comput Healthc.

[41] Urbanczyk T, Peter L (2016). Database development for the urgent department of hospital based on tagged entity storage following the IoT concept. Proceedings of the 14th IFAC Conference on Programmable Devices and Embedded Systems PDES 2016.

[42] Shahid J, Ahmad R, Kiani AK, Ahmad T, Saeed S, Almuhaideb AM (2022). Data protection and privacy of the internet of healthcare things (IoHTs). Appl Sci.

[43] Iliev IT, Badarov DH, Tabakov SD, Ganev BT, Kanev IK (2020). Fully analogue ECG front-end applicable in remote patient monitoring. Proceedings of the XXIX International Scientific Conference Electronics (ET).

[44] Fersi G (2020). Study of middleware for Internet of healthcare things and their applications. Proceedings of the International Conference on Smart Homes and Health Telematics.

[45] Vishnu S, Ramson SR, Jegan R (2020). Internet of medical things (IoMT)- an overview. Proceedings of the 5th International Conference on Devices, Circuits and Systems (ICDCS).

[46] Venkatesha S, Reddy KR, Chandavarkar BR (2021). Social engineering attacks during the COVID-19 pandemic. SN Comput Sci.

[47] Leonov PY, Vorobyev AV, Ezhova AA, Kotelyanets OS, Zavalishina AK, Morozov NV (2021). The main social engineering techniques aimed at hacking information systems. Proceedings of the Ural Symposium on Biomedical Engineering, Radioelectronics and Information Technology (USBEREIT).

[48] Gupta S, Singhal A, Kapoor A (2016). A literature survey on social engineering attacks: phishing attack. Proceedings of the International Conference on Computing, Communication and Automation (ICCCA).

[49] Gan D, Heartfield R (2016). Social engineering in the internet of everything. Cut IT J.

[50] Ingle DR, Meshram BB (2012). Attacks on web based software and modelling defence mechanisms. Int J UbiComp.

[51] Fang L, Li Y, Yun X, Wen Z, Ji S, Meng W, Cao Z, Tanveer M (2020). THP: a novel authentication scheme to prevent multiple attacks in SDN-based IOT network. IEEE Internet Things J.

[52] Hassan MM, Nipa SS, Akter M, Haque R, Deepa FN, Rahman MM, Siddiqui M, Sharif MH (2018). Broken authentication and session management vulnerability: a case study of web application. Int J Simul Syst Sci Technol.

[53] Nadar VM, Chatterjee M, Jacob L (2017). A defensive approach for CSRF and broken authentication and session management attack. Proceedings of the International Conference on Recent Advancements in Computer, Communication and Computational Sciences (RACCCS-2017).

[54] Wang D, Wang P (2013). Offline dictionary attack on password authentication schemes using smart cards. Proceedings of the 16th International Conference, ISC 2013.

[55] Dewangan K, Mishra M (2018). Internet of things for healthcare: a review. Int J Adv Manag Technol Eng Sci.

[56] Li C-T, Shih D-H, Wang C-C (2018). Cloud-assisted mutual authentication and privacy preservation protocol for telecare medical information systems. Comput Methods Programs Biomed.

[57] Shim K-A (2019). Universal forgery attacks on remote authentication schemes for wireless body area networks based on internet of things. IEEE Internet Things J.

[58] Soni M, Patel T, Jain A (2018). Security analysis on remote user authentication methods. Proceeding of the International Conference on Computer Networks, Big Data and IoT (ICCBI - 2018).

[59] Shaukat HR, Hashim F, Sali A, Abdul Rasid MF (2014). Node replication attacks in mobile wireless sensor network: a survey. Int J Distrib Sens Netw.

[60] Manjula V, Chellappan C (2011). The replication attack in wireless sensor networks: analysis and defenses. Proceedings of the International Conference on Computer Science and Information Technology.

[61] Mishra AK, Tripathy AK, Puthal D, Yang LT (2019). Analytical model for Sybil attack phases in internet of things. IEEE Internet Things J.

[62] Bošnjak L, Sreš J, Brumen B (2018). Brute-force and dictionary attack on hashed real-world passwords. Proceedings of the 41st International Convention on Information and Communication Technology, Electronics and Microelectronics (MIPRO).

[63] Salamatian S, Huleihel W, Beirami A, Cohen A, Medard M (2019). Why botnets work: distributed brute-force attacks need no synchronization. IEEE Trans Inform Forensic Secur.

[64] Singh P, Basit A, Kumar NC, Venkaiah VC (2023). Towards a hybrid Public Key Infrastructure (PKI): a review. Cryptology ePrint Archive. Preprint posted online July 14, 2019.

[65] Nag AK, Dasgupta D (2014). An adaptive approach for continuous multi-factor authentication in an identity eco-system. Proceedings of the 9th Annual Cyber and Information Security Research Conference.

[66] Muhammad G, Alqahtani S, Alelaiwi A (2021). Pandemic management for diseases similar to COVID-19 using deep learning and 5G communications. IEEE Netw.

